# Effect of Delayed Irrigation at the Jointing Stage on Nitrogen, Silicon Nutrition and Grain Yield of Winter Wheat in the North China Plain

**DOI:** 10.3390/plants13182648

**Published:** 2024-09-21

**Authors:** Hao Zheng, Jinyang Sun, Yueping Liang, Caiyun Cao, Yang Gao, Junpeng Zhang, Hongkai Dang, Chunlian Zheng

**Affiliations:** 1 Shandong Key Laboratory of Agricultural Water-Saving Technology and Equipment, College of Water Conservancy and Civil Engineering, Shandong Agricultural University, Tai’an 271018, China; 17866702681@163.com (H.Z.); sunjinyang123456@126.com (J.S.); jpengzhang@163.com (J.Z.); 2 Hebei Provincial Key Laboratory of Crop Drought Resistance Research, Institute of Dryland Farming, Hebei Academy of Agriculture and Forestry Sciences, Hengshui 053000, China; cycao1234@126.com; 3 Institute of Farmland Irrigation, Chinese Academy of Agricultural Sciences, Xinxiang 453000, China; yueping0520@163.com (Y.L.); gaoyang@caas.cn (Y.G.)

**Keywords:** wheat, grain yield, nitrogen, silicon, water-saving irrigation

## Abstract

Water scarcity is a key limitation to winter wheat production in the North China Plain, and it is essential to explore the optimal timing of spring irrigation to optimize N and Si uptake as well as to safeguard yields. The aim of this study was to systematically study the effect mechanism of nitrogen and silicon absorption of winter wheat on yield under spring irrigation and to provide a scientific basis for optimizing irrigation strategy during the growth period of winter wheat. In this experiment, the winter wheat ‘Heng 4399’ was used. Five irrigation periods, i.e., 0 d (CK), 5 d (AJ5), 10 d (AJ10), 15 d (AJ15), and 20 d (AJ20) after the jointing stage, were set up to evaluate the nitrogen (N) and silicon (Si) absorption and grain yield (GY). The results showed that delayed irrigation for 5–10 days at the jointing stage had increased the GY. With the delay of irrigation time, the N/Si content of the entire plant at the maturity period increased first and then decreased; among that, the maximum N contents appeared in AJ15 and AJ5 in 2015 and 2020, respectively, while the Si concentrations appeared in AJ5 and AJ10 in sequence. Compared with AJ15 and AJ20, the N accumulation of vegetative organs in AJ5 increased by 3.05~23.13% at the flowering stage, 14.12~40.12% after the flowering stage, and a 1.76~6.45% increase in the N distribution rate at maturity stage. A correlation analysis revealed that the GY was significantly and positively correlated with the N/Si accumulation at the anthesis and N translocation after the anthesis stage. In conclusion, under limited irrigation conditions, delaying watering for 5 to 10 days at the jointing stage can improve the nitrogen and silicon absorption and nutrient status of wheat plants and increase wheat yield.

## 1. Introduction

Winter wheat is one of the main grain crops in the North China Plain (NCP) [1], which accounts for about 70% of the total wheat production in China [2]. In the NCP, the maize-in-summer/wheat-in-winter rotation system is common under the constraint of precipitation deficit, which is far less than the amount required for normal growth and development of winter wheat during the growing season [3,4,5], where two irrigations in spring after sowing are needed for high yield [6]. Since the 1970s, groundwater as the main source for crop irrigation has been increasingly depleted from the groundwater table, causing a series of geological disasters [7]. Therefore, implementing a more efficient irrigation strategy and technology in the region is imperative to conserve water while achieving high and stable yield.

Deficit irrigation is a water-saving irrigation strategy in which less than the full water requirement of the crop is applied only during critical growth stages. Extensive research has shown that an appropriate reduction of irrigation can increase grain yield and water use efficiency [8]. Zeng et al. [2] showed that the water productivity of winter wheat increased significantly when irrigation was reduced by 50%, while the difference in yield was minor. The jointing stage is a critical period of water demand for wheat, and sufficient water at this stage can significantly increase yield [9]. However, a mild water deficit before the jointing stage did not negatively affect wheat yield but slightly increased it [10]. Fan et al. [11] reported that delayed irrigation during the jointing stage could effectively improve photosynthetic capacity and water use efficiency, resulting in increased yield, while Li et al. [12] showed that the high yield of winter wheat can be maintained by irrigating once in spring.

Deficit irrigation will cause drought stress to wheat, and it is a crucial action for wheat to take measures to reduce the adverse effects of water shortage. Nitrogen (N) is one of the main factors limiting the productivity of various cereal crops, including wheat [13,14,15]. Nitrogen is involved in various key processes, such as leaf expansion, biomass accumulation, and plant height [16]. The application of a high nitrogen supply promoted the transport of carbohydrates from vegetative organs to panicles at the late growth stage [17]. More and more studies have shown that nitrogen nutrition is essential to improving plant stress resistance. Reasonable nitrogen management can alleviate the negative effects of drought on wheat yield formation and resource utilization efficiency. Yan et al. [18] found that with the increase in water stress, the total nitrogen uptake decreased, but there was no significant difference between normal irrigation and mild deficit irrigation. Even Yong et al. [19] thought that a certain degree of water stress could promote the absorption of nitrogen. The potential of silicon (Si) to enhance environmental stress tolerance has been confirmed [20]. In the mineral elements found in different crops, silicon content reached 0.1%~10.0% of plant dry weight [21]. Silicon can reduce the destructive effects of biotic [22] and abiotic [23] stresses in wheat plants. It plays a vital role in plant growth by enhancing mineral nutrition and mechanical strength and improving plant resistance to abiotic stresses [24]. Nadeem et al. [25] found that silicon can enhance the performance of wheat in reverse environments such as drought, saline-alkali, and nutrient deficit conditions. Silicon-induced plant resistance to stress may be attributed to the formation of plant rocks and the polymerization of insoluble silicon around cell walls and vascular bundles, reducing water loss by controlling evaporation [26]. Therefore, silicon is one of the important elements required for wheat to maintain a high yield [27]. However, Salem [28] showed that a decrease in irrigation water leads to a decrease in plant silicon content.

At present, the growth [29] and photosynthetic characteristics [30] of winter wheat under different stages of spring irrigation have been well understood through previous research. However, the research on the accumulation and distribution of N and Si in winter wheat plants varied according to geographical location, climatic conditions, and other factors. Therefore, this paper aimed to compare the effects of spring irrigation at the critical jointing stage on the accumulation and distribution of N and Si and the GY of winter wheat between a normal (2014–2015) and a dry (2019–2020) year in the NCP. It will provide a theoretical basis for the water-saving and high-yield cropping strategy.

## 2. Materials and Methods

### 2.1. Experimental Sites

The field experiment was conducted during the 2014–2015 and 2019–2020 wheat seasons in a site located in the Hengshui Experimental Station of Dry-land Farming Research Institute, Hebei Academy of Agricultural and Forest Sciences (115°72′ E, 37°91′ N), Shenzhou County, Hengshui, Hebei Province in the NCP. The experimental field has a semi-humid and semi-arid climate with an annual average temperature of 12.8 °C, an annual evaporation rate of 1785 mm, an annual sunshine duration of 2509.4 h, an annual frost-free period of 188 days, and an annual precipitation of 510 mm. The soil of the experimental site was loamy with a soil bulk density of 1.46 g·cm^−3^. The nutrient contents in topsoil (20 cm) had 15.66~17.42 g·kg^−1^ organic matter, 1.48~1.54 g·kg^−1^ total N, 127.2~134.68 mg·kg^−1^ available N, 21.95~24.13 mg·kg^−1^ available P, and 113.68~128.42 mg·kg^−1^ available K.

**Figure 1:**
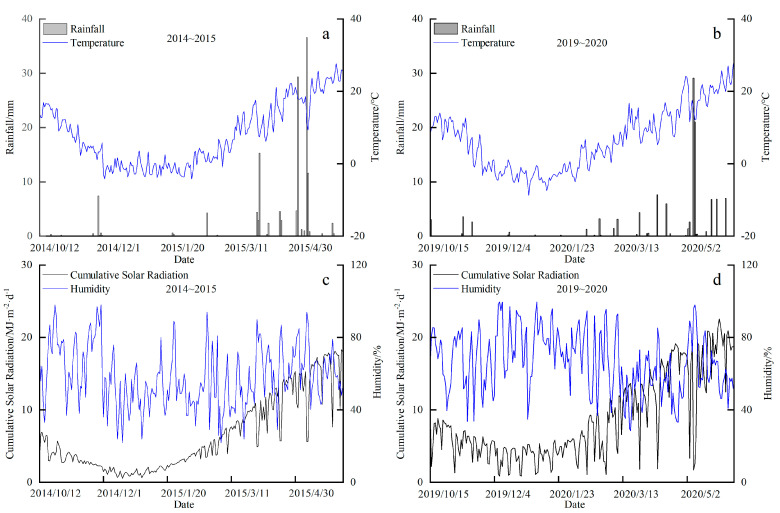
Meteorological map of winter wheat growth period. Note: The subfigures (**a**,**b**) represent the daily rainfall and average temperature in 2014–2015 and 2019–20202, respectively. The subfigures (**c**,**d**) represent the daily cumulative solar radiation and relative humidity in 2014–2015 and 2019–2020, respectively.

The climatic year type was divided by the classification method of annual crop climate types: $∆R=\frac{X-X_{p}}{s}$, in which ∆R is the anomaly percentage distance, X is the rainfall during the whole growth period of crops, $X_{p}$ is the average value of rainfall elements, and s is the standard deviation. When ∆R ≥ 0.3, it is a wet year; when ∆R ≤ −0.3, it is a dry year; and when −0.3 ≥ ∆R ≥ 0.3, it is a normal year. According to the calculation, the values of ΔR in 2014–2015 and 2019–2020 are −0.08 and −1.09, respectively, which belong to the normal year and the dry year. The meteorological data during two wheat-growing seasons are presented in Figure 1 and Table 1.

### 2.2. Experimental Design

The winter wheat variety planted in the field experiment was ‘Heng 4399‘. The winter wheat-summer maize double cropping system was adopted in one year. After the maize was harvested, the straw was all crushed and returned to the field. Winter wheat was sown on 12 October 2014 and 15 October 2019, respectively, and harvested on 6 June 2015 and 7 June 2020, respectively. The sowing rate was 225 kg·hm^−2^ with a row spacing of 15 cm. The plots were irrigated with 75 mm of water before sowing to ensure seed germination and emergence. Urea, diammonium hydrogen phosphate, and potassium chloride were applied at 375 kg·hm^−2^ and 150 kg·hm^−2^, respectively, equivalent to 267 kg·hm^−2^ N, 241.5 kg·hm^−2^ P_2_O_5_, and 90 kg·hm^−2^ K_2_O. The field experiment was arranged in a randomized complete block design with three replications for each treatment. During the critical jointing period, 5 treatments of delayed irrigation days were set as follows: 0d (CK, AJ0), 5d (AJ5), 10d (AJ10), 15d (AJ15), and 20d (AJ20) after the start of jointing. In AJ0, 75 mm irrigation was used as a control. The individual plot was 40 m^2^ (5 m × 8 m) with a 0.5 m buffer between plots.

### 2.3. Date Collection and Calculation

#### 2.3.1. Plant Biomass

Aboveground plant samples (n = 30) were randomly collected at the jointing stage (JS), heading stage (HS), anthesis stage (AS), filling stage (FS), and maturity stage (MS) of winter wheat. Plant samples at anthesis and maturity were separated into three components (stem/sheath, leaf, and spike). The samples were dried in an oven at 80 °C for 72 h after heating at 105 °C for 30 min and then ground. Finally, N and Si contents were measured in different parts of the winter wheat.

#### 2.3.2. N Content Determination

Dried samples from all growth stages were ground and the dry matter was measured, and the N content of each sample was calculated by digestion of a 0.25 g sample in sulfuric acid (H_2_SO_4_) and hydrogen peroxide (H_2_O_2_) and measured using the Kjeldahl method [31]. The N content in different parts of the wheat plant was determined by multiplying the total dry matter of each plant organ by the N content and calculated in kg·hm^−2^.

(1) NAA=aboveground dry matter accumulation×N content

(2) $${NTA}_{stem}={NAAA}_{stem}-{NAAM}_{stem}$$

(3) $${NTA}_{leaf}={NAAA}_{leaf}-{NAAM}_{leaf}$$

(4) $${{NTA}_{spike}={NAAA}_{spike}-NAAM}_{spike}$$

(5) $$N\;distribution\;rate\;in\;each\;organ=\frac{N\;accumulation\;in\;each\;organ}{total\;N\;accumulation}\times 100\%$$

#### 2.3.3. Si Content Determination

The incinerated samples and their extraction were performed according to the method described by Hogendorp [32], and the colorimetric determination was calculated according to the method of Yang [33]:

(6) $${SiO}_{2}\%=\frac{Microgram\;number\;of\;solution\times Color\;volume\times Dividing\;multiple}{Sample\;weight\times 10^{4}}\times 100$$

(7) SiAA=aboveground dry matter accumulation×Si content

(8) $${SiAAAM}_{stem}={SiAAA}_{stem}-{SiAAM}_{stem}$$

(9) $${SiAAAM}_{leaf}={SiAAA}_{leaf}-{SiAAM}_{leaf}$$

(10) $${SiAAAM}_{spike}={SiAAA}_{spike}-{SiAAM}_{spike}$$

(11) $$Si\;distribution\;rate\;in\;each\;organ=\frac{Si\;accumulation\;in\;each\;organ}{total\;Si\;accumulation}\times 100\%$$

Formula: NAA: N accumulation amount; NTA_stem_: N translocation amount from stem and sheath; NAAA_stem_: N accumulation amount in stem and sheath at anthesis; NAAM_stem_: N accumulation amount in stem and sheath at maturity; NTA_leaf_: N translocation amount from leaf; NAAA_leaf_: N accumulation amount in leaf at anthesis; NAAM_leaf_: N accumulation amount in leaf at maturity; NTA_spike_: N translocation and accumulation amount to the ear; NAAA_spike_: N accumulation amount in ear at anthesis; NAAM_spike_: N accumulation amount in ear at maturity; SiAA: Si accumulation amount; SiAAAM_stem_: Si accumulation amount in stem and sheath from anthesis to maturity; SiAAA_stem_: Si accumulation amount in stem and sheath at anthesis; SiAAM_stem_: Si accumulation amount in stem and sheath at maturity; SiAAAM_leaf_: Si accumulation amount in leaf from anthesis to maturity; SiAAA_leaf_: Si accumulation amount in leaf at anthesis; SiAAM_leaf_: Si accumulation amount in leaf at maturity; SiAAAM_spike_: Si accumulation amount in ear from anthesis to maturity; SiAAA_spike_: Si accumulation amount in ear at anthesis; SiAAM_spike_: Si accumulation amount in ear at maturity.

#### 2.3.4. Grain Yield

Grain yield (GY) was determined by harvesting the wheat ears in the middle rows of each treatment plot. Winter wheat plants in 1 m^2^ were harvested as one of three replicates of each treatment for the calculation of GY.

### 2.4. Statistical Analyses

The experimental data were collected and analyzed using Excel 2021 and plotted using the R-4.3.3 program and Origin 2021. Multiple comparisons were performed using SPSS 26.0 software, and the significance testing was performed using LSD with 0.05 as the level of significant difference.

## 3. Results

### 3.1. Changes of N and Si Contents in Winter Wheat and Their Distribution in Various Organs

#### 3.1.1. Dynamics of the N and Si Content in Winter Wheat

The N contents at different growth stages were 12.41~34.48 mg·g^−1^ (Table 2), and the contents in different treatments in the same growing season decreased continuously. At the maturity stage, the N contents in 2015 and 2020 were 14.21~15.98 mg·g^−1^ and 12.54~13.43 mg·g^−1^, respectively. There was no significant difference in N content between treatments at maturity in 2015. The treatment with the highest N content in 2020 was AJ5, and there was little difference between AJ10 and AJ20, but significantly higher than CK and AJ20. In the same year, the treatments with the highest N content required for 100 kg grain construction were CK and AJ15 in 2015 and 2020, respectively, and the treatment with the lowest N content was AJ10. The Si content in winter wheat with different treatments was 7.83~19.27 mg·g^−1^. At maturity, the Si content was highest in AJ5 in 2015, 7.80%~10.40% higher than that in AJ10, AJ15, and AJ20. The highest Si content was recorded in AJ10 in 2020, which was not significantly different from AJ15, but significantly higher than the other treatments. The treatment with the greatest Si accumulation in 2015 was CK, which was significantly greater than AJ15 and AJ20 with an increase of 10.67~11.48%. AJ10 with the highest Si accumulation in 2020 was significantly greater than the other treatments with an increase of 15.03~39.19%.

#### 3.1.2. Content and Accumulation of N and Si in Different Parts

**Figure 2:**
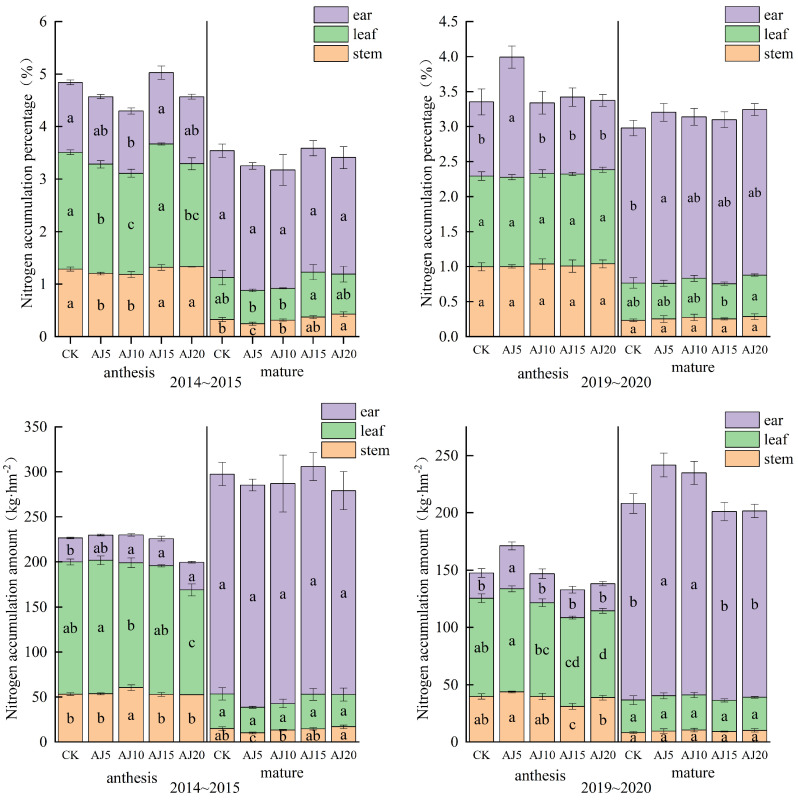
The N contents and accumulations in different wheat parts at the anthesis and maturity stage. Note: Values within columns followed by the same letter are statistically insignificant at the 0.05 level.

In different parts of wheat at anthesis, the N content is shown as ear > leaf > stem, while the N accumulation showed leaf > stem > ear with more delayed irrigation time (Figure 2). The N accumulation in each part showed an increasing and then decreasing trend. At the anthesis stage, the stem and leaf obtained the maximum N accumulation in AJ10 and AJ5 in 2015, respectively. In 2020, the N accumulation of stem and leaf reached the maximum in AJ5 with an increase of 9.63~40.14% and 5.04~18.84%, respectively. At the maturity stage, the N contents and accumulations in different parts of wheat showed a trend of spike > leaf > stem, and the N accumulation in spike reached the maximum in AJ15 and AJ5 in the two years. Between them, the difference in the N accumulation was not significant in 2015, but in 2020, the difference between AJ5 and AJ10 was not significant but significantly greater than other treatments.

**Figure 3:**
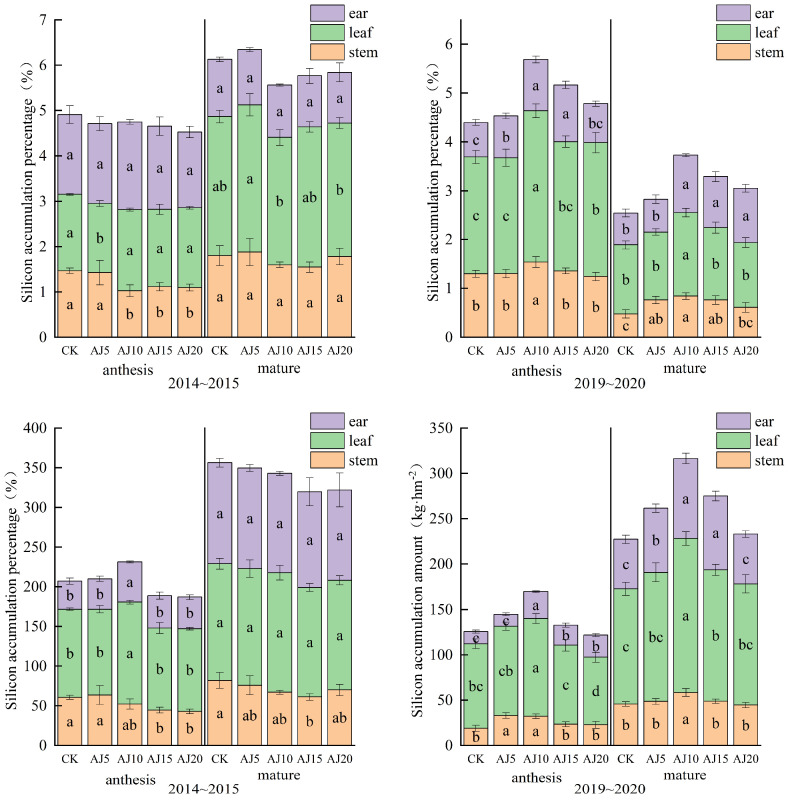
The Si contents and accumulations in different wheat parts at the anthesis and maturity stage. Note: Values within columns followed by the same letter are statistically insignificant at the 0.05 level.

The contents of Si in different parts at anthesis were higher in leaf and spike than in stems, and the trend of Si accumulation is manifested as leaf > stem > spike (Figure 3). With more delayed irrigation time, the Si accumulation in each wheat part showed a trend of first increasing and then decreasing. At the anthesis stage, Si accumulation in stems reached the maximum in AJ5 in both years, and Si accumulation in leaves and spikes reached the maximum in AJ10. At the maturity stage with the delayed irrigation time, the Si accumulation in stems showed a trend of first decreasing and then increasing in 2015, and the maximum value was obtained in CK. The difference in Si accumulation between leaves and spikes was not significant. In 2020, all the wheat parts showed a trend of increasing and then decreasing. The Si contents in stems, leaves, and spikes reached the maximum in AJ10, AJ10, and AJ15 with an increase of 13.13~23.43%, 13.18~30.93%, and 10.42~64.83%, respectively, compared to other treatments.

#### 3.1.3. Distribution of N and Si in Different Wheat Parts

The N distribution rate of N in leaves was 58.4~64.8% at anthesis and decreased to 10.1~14.4% at maturity (Table 3). The distribution rate of N in stems decreased from 23.4~28.4% at the anthesis stage to 3.5~6.1% at the maturity stage. The N distribution rate in ears gradually increased from 11.8~19.8% at the anthesis stage to 80.6~86.4% at the maturity stage. The N distribution rates in stems and leaves of different treatments in the two-year maturity period showed a trend of first decreasing and then increasing, and the N distribution rate in spikes showed a trend of first increasing and then decreasing. In 2015 and 2020, the N distribution rates in spikes reached the maximum in AJ5 with an increase of 2.73~6.40% and 0.97~3.35% compared to other treatments, respectively.

At the anthesis stage, Si distribution rates were 51.5~74.3%, 15~30.2%, and 9~21.9% in leaves, stems, and spikes, respectively. At the maturity stage, the Si distribution rate decreased to 41.3~57.3% in leaves, remained unchanged at 17.6~23% in stems, and increased to 23.6~37.6% in spikes. At the maturity stage, there was no significant difference in the Si distribution rates between different treatments in 2015. In 2020, the Si distribution rate showed a trend of first increasing and then decreasing with a maximum value in AJ15 that was significantly greater than other treatments.

#### 3.1.4. Net Uptake and Transfer of N and Si in Different Organs

The Table 4 shows that stems and leaves started to transfer N after flowering, and the transferred amounts were 22.02~47.10 kg·hm^−2^ and 46.83~119.34 kg·hm^−2^, respectively. The amount transferred by leaves was the highest in both years. In 2015 and 2020, the N contribution rate from stems and leaves to ears reached about 50%, indicating that N uptake and accumulation largely depended on the N redistribution from vegetative organs. Between the different irrigation treatments and with delayed irrigation at the jointing stage, the total amounts of N transferred from stems and leaves showed a trend of first increasing and then decreasing, reaching a peak amount at AJ5 or AJ10 in both years. The Si content of each wheat part increased from the flowering to the maturing stage, and the trend of Si accumulation in different wheat parts was ear > leaf > stem. The amount of Si absorbed by spikelet in 2015 was much higher in 2015 than in 2020. In 2015, the Si uptake of each wheat part was not significantly different. In 2020, Si uptake in stems, leaves, and ears was maximum in CK, AJ10, and AJ15, respectively.

### 3.2. GY and Its Correlation with the N and Si Accumulation

#### 3.2.1. Impact of Irrigation Duration and Different Years on the GY and Irrigation Water Use Efficiency of Winter Wheat

**Figure 4:**
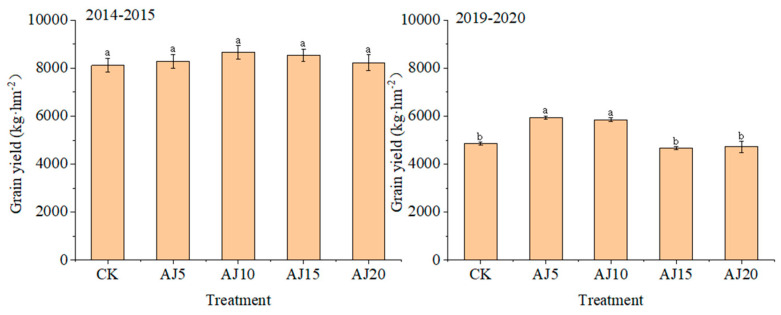
Grain yield of winter wheat. Mean values in a separate column followed by similar letters were not significantly different at *p* < 0.05. The values are the means ± SE (standard error).

**Figure 5:**
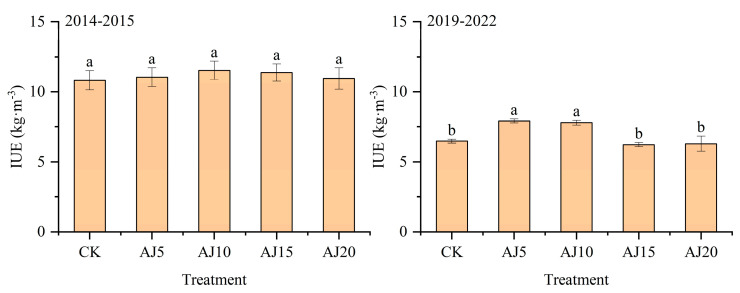
IUE of winter wheat. Mean values in a separate column followed by similar letters were not significantly different at *p* < 0.05. The values are the means ± SE (standard error).

In the two experimental years, the GY and irrigation water use efficiency (IWUE) of winter wheat first increased and then decreased with delayed irrigation time (Figure 4 and Figure 5). Among them, the GY of winter wheat reached the maximum in AJ10 with an increase of 1.37~6.57% compared with other treatments in 2015. Compared with CK and other treatments, the GY in AJ5 and AJ10 increased significantly by 22.12~27.20% in 2020, and the difference between AJ5 and AJ10 was not significant. The GY of winter wheat in 2020 was much lower than that in 2015. Compared with 2020, the GY of each treatment decreased by 28.25~45.25% than that in 2015. The IWUE was consistent with the change rule of yield.

The F-value test (Table 5) showed that irrigation time (I) had a very significant effect on GY, NAAA, NTA_stem_, NTA_leaf_, SiAAA, SiAAM, while the different year (Y) had a very significant effect on GY, NAAA, NAAM, NTA_stem_, NTA_leaf_, SiAAA, SiAAM. The interaction between them (I × Y) had a significant effect on GY, NAAM, NTA_leaf_, and a most significant effect on NAAA, NTA_stem_, and SiAAM.

#### 3.2.2. Relationship between the Studied Parameters

**Figure 6:**
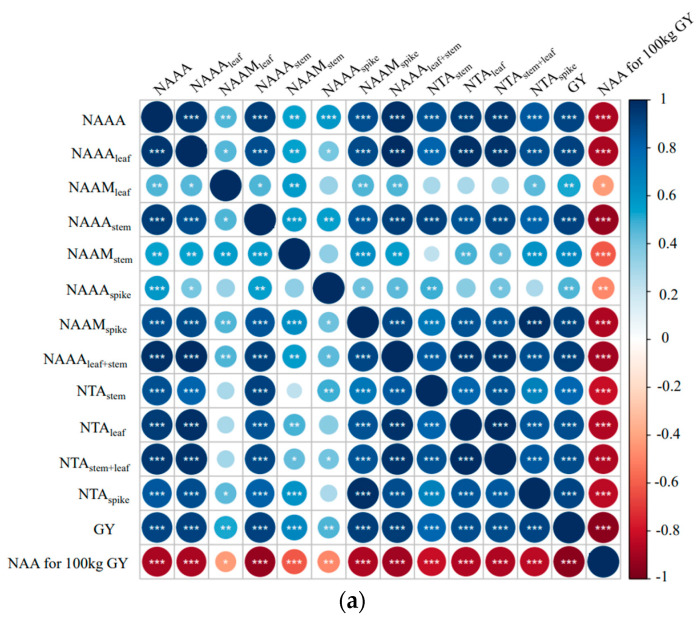

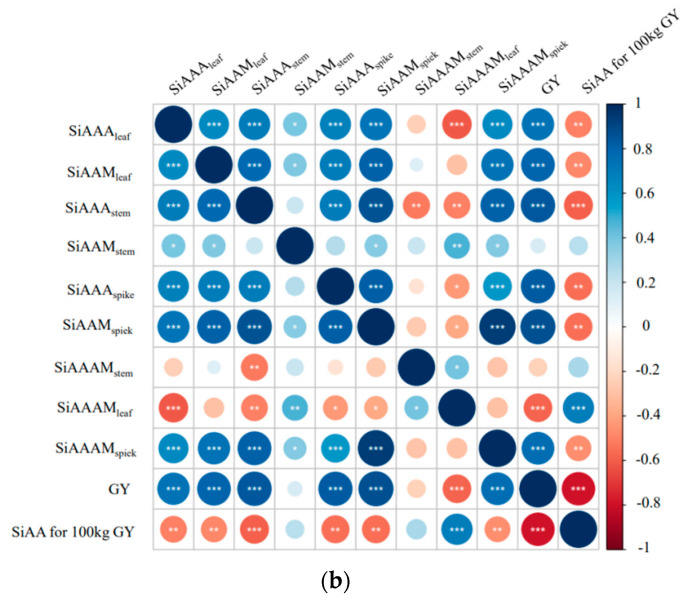
The relationship between the yield and other studied parameters. (**a**) Correlation analysis between grain yield and N accumulation and translocation; (**b**) Correlation analysis between grain yield and silicon accumulation and translocation. Note: The intensity of color represents the significance of a variable. Blue represents a positive correlation, and red represents a negative correlation. *, **, *** significant at the 0.05, 0.01, and 0.001 probability levels, respectively. GY: grain yield; NAAA: N accumulation amount at anthesis; NAAM: N accumulation amount at maturity; NAAA_leaf_: N accumulation amount in leaf at anthesis; NAAM_leaf_: N accumulation amount in leaf at maturity; NAAAstem: N accumulation amount in stem and sheath at anthesis; NAAM_stem_: N accumulation amount in stem and sheath at maturity; NAAA_spike_: N accumulation amount in ear at anthesis; NAAM_spike_: N accumulation amount in ear at maturity; NAAA_stem+ leaf_: N accumulation amount in stem, sheath, and leaf at anthesis; NTA_stem_: N translocation amount from stem and sheath; NTAleaf: N translocation amount from leaf; NTAspike: N translocation and accumulation amount to the ear; NAA for 100 GY: N accumulation amount for per 100 kg grain yield construction; SiAAA: Si accumulation amount at anthesis; SiAAM: Si accumulation amount at maturity; SiAAA_leaf_: Si accumulation amount in leaf at anthesis; SiAAM_leaf_: Si accumulation amount in leaf at maturity; SiAAA_stem_: Si accumulation amount in stem and sheath at anthesis; SiAAM_stem_: Si accumulation amount in stem and sheath at maturity; SiAAAspike: Si accumulation amount in ear at anthesis; SiAAMspike: Si accumulation amount in ear at maturity; SiAAAM_stem_: Si accumulation amount in stem and sheath from anthesis to maturity; SiAAAM_leaf_: Si accumulation amount in leaf from anthesis to maturity; SiAAAM_spike_: Si accumulation amount in ear from anthesis to maturity; SiAA for 100 GY: Si accumulation amount for per 100 kg grain yield construction.

Correlation analysis of GY with N and Si accumulation and translocation (Figure 6) showed that it was significantly and positively correlated with NAAA, NAAA_leaf_, NAA_meaf_, NAAA_stem_, NAAN_stem_, NAAA_stem+leaf_, NAAA_spike_, NAAM_spike_, NTA_stem_, NTA_leaf_, NTA_stem+leaf_, and NTA_spike_, but negatively correlated with NAA for 100 kg GY. At the same time, NAAA_stem_ and NAAA_leaf_ were positively correlated with the amount of N translocated from different parts of the wheat after flowering. In this experiment, delayed irrigation during the jointing stage increased the N accumulation in vegetative organs at the anthesis stage, increased the N transfer from vegetative organs after the anthesis stage, and increased the N accumulation in grains for higher GY. GY was found to be positively correlated with SiAAA_leaf_, SiAAM_leaf_, SiAAA_stem_, SiAAA_spike_, SiAAM_spike_, and SiAAAM_spike_, and negatively correlated with SiAAAM_leaf_ and SiAA for 100 kg GY, indicating that increasing Si content in stems and ears promotes GY to some extent.

## 4. Discussion

### 4.1. The Relationship between the N and Si Content, Accumulation, and Yield Level of Winter Wheat

Our research showed that the N contents in the whole plant at the maturity stage of winter wheat were 1.42~1.6% and 1.25~1.34% in 2015 and 2020, respectively, which was slightly lower than the results (2.10~3.24%) of Li [34]. The Si content was 1.67~1.84% and 1.37~1.79% in both years, respectively, which was lower than that determined by Li [35] in wheat straw (2.5~5%). At the yield level of this study, the highest N accumulation in both years was 201.15~305.97 kg·hm^−2^, which was similar to 200~400 kg·hm^−2^ determined by Liang [36] at the yield level of 7881~9588 kg·hm^−2^ at the maturity stage. The highest Si accumulation was 227.44~356.35 kg·hm^−2^, which was higher than 115.42~159.24 kg·hm^−2^ determined by He [37] at the yield level of 3673.47~5102.04 kg·hm^−2^. The difference may be attributed to the light quality and quantity, cultivation pattern, soil texture, and other parameters.

In 2015 and 2020, the N uptake per 100 kg grain was 3.35~3.67 kg and 4.01~4.31 kg, respectively. He et al. [38] showed that under the yield level of 7387~10,414 kg·hm^−2^, the total N accumulation was 151~256 kg·hm^−2^, and the N uptake per 100 kg grain was 3.35~5.15 kg. Li et al. [39] proved that under the yield level of 5161~7671 kg·hm^−2^, the total N accumulation was 193~290 kg·hm^−2^, and the N uptake per 100 kg grain was 3.53~3.78 kg. With the increase of GY per unit area, the total N uptake of wheat increased significantly (r = 0.950, *p* < 0.001), while the N uptake per unit GY decreased (r = −0.959, *p* < 0.001), which was consistent with the result of previous research [40]. The Si uptake per 100 kg grain ranged from 3.75 to 4.39 kg and 4.40 to 5.89 kg in 2015 and 2020, respectively. With the increase in yield, the total Si uptake had an increasing trend (r = 0.773, *p* < 0.001), and the Si uptake per 100 kg of grains had a decreasing trend (r = 0.800, *p* < 0.001). In conclusion, we found that under the condition with different delayed spring irrigation times, the amount of the N and Si uptake per 100 kg of grains decreased, and the N/Si utilization efficiency for the grain production was improved to increase the GY.

### 4.2. N Nutritional Characteristics of Winter Wheat and Its Effect on GY under Different Times of Spring Irrigation

Water affects N accumulation and transport in crops [41]. Studies have shown that N accumulation in wheat increased with more irrigation water [42]. Water deficiency from the jointing to anthesis stage has the greatest impact on N absorption [43,44]. However, slight water stress can be beneficial to increase the N accumulation in wheat [19]. The jointing stage is a key period for water demand in winter wheat [9]. Delayed jointing irrigation has been shown to promote N accumulation in vegetative organs and its transport to grains, and to increase N accumulation after the anthesis stage and N partitioning ratio in grains at the maturity stage [45]. With a delay in irrigation time, our data showed that N accumulation in each wheat part tended to first increase and then decrease. At the anthesis stage, N accumulation in stems and leaves reached the maximum amount in AJ10 and AJ5 in 2015, respectively. In 2020, N accumulation in both leaves and stems reached the maximum amount in AJ5, while at the maturity stage, N accumulation in ears reached the maximum amount in AJ15 and AJ5 in both years. Therefore, the timing of irrigation may affect the efficiency of water and available N uptake by roots [46,47]. Early irrigation may lead to insufficient water supply later, resulting in reduced N uptake from the soil due to reduced nutrient diffusion and massive leakage in the soil [28]. The Lu et al. [48] study found that water shortage would reduce the nitrogen accumulation of winter wheat by 23.2%. However, if irrigation is applied too late, it can lead to a water shortage at the critical stage, and water cannot be fully utilized. Combined with previous studies on water use [29,34], we found that whole-plant N was highly correlated with water use (r = 0.820, *p* < 0.001). In addition, in this experiment, supplemental N fertilization with irrigation met the second N requirement of wheat and promoted GY [49]. During the two years, the yield reached the maximum in AJ10 and AJ5, respectively, which increased by 6.57%, 1.37%, 5.27%, and 22.12%, 27.21%, and 25.86% compared with CK, AJ15, and AJ20, respectively. However, much delayed irrigation may be affected by more rainfall in the late growth stage, and some N fertilizer may be lost through guttation, root secretion, and rain leaching [50], which in turn inhibits N accumulation during flowering. The statistical analysis of this study showed that GY was significantly and positively correlated with N accumulation in vegetative parts at anthesis, N translocation after anthesis, and N accumulation in spikes at maturity. It suggested that appropriate delayed irrigation at the jointing stage could promote N accumulation after anthesis and the N partitioning ratio in the ear at the maturing stage by promoting N accumulation in vegetative organs and its transport to grains for high yield.

### 4.3. Si Nutritional Characteristics of Winter Wheat and Its Effect on the GY under Different Irrigation Times in Spring

Si can increase the tolerance of plants to environmental stresses [25]. Previous studies have shown that exogenous Si could significantly improve the growth and development of winter wheat under drought stress [51], and it was generally believed that there were differences in the distribution of Si in plants, which generally followed the end-distribution [52], where the highest Si content was in leaves and the lowest in roots. This experiment confirmed that Si in wheat followed the end distribution and that the highest accumulation occurred mainly in leaves. The accumulation of silicon in leaves is beneficial to the growth and yield of winter wheat. The results showed that Si had a beneficial effect on the greening of leaves and could improve the chlorophyll content. In addition, Si could reduce the stomatal conductance and water loss of leaves [53,54]. In this experiment, compared with CK, AJ15, and AJ20 treatments, the leaf silicon accumulation of AJ10 treatment at the flowering stage increased by 15.56~44.67%, and the yield increased by 1.37~23.93%, which was consistent with the above conclusions. In addition, this study has shown that the Si content in the whole plant in 2015 and 2020 showed a trend of first increasing and then decreasing, and the variation range of silicon accumulation in each treatment was 319.64~356.35 kg·hm^−2^ and 227.44~346.56 kg·hm^−2^, respectively. The highest amount is in AJ5 and AJ10, respectively. There was also a significant difference in Si content between years (F = 10.038, *p* = 0.004), as Si absorption is water dependent [28]. Appropriately delayed irrigation brings soil moisture in line with the water demand and promotes water uptake by wheat [29]. Combined with previous studies on water use [28,33], we found that whole-plant Si accumulation was highly correlated with water use (r = 0.762, *p* < 0.001). With delayed irrigation time, Si partitioning rates of winter wheat in leaves and ears first increased and then decreased. Correlation analysis showed that GY was significantly and positively correlated with Si content in each of the wheat organs at the anthesis stage and its content in ears at the maturity stage. These results indicate that suitable delayed irrigation at the jointing stage can promote the proportion of silicon in the growth center of leaves and ears, improve stress resistance, and then affect the higher GY.

### 4.4. Effect of Different Spring Irrigation Times on the IUE of Winter Wheat

Moderate water stress can improve the irrigation water utilization efficiency of wheat [55]. Through the analysis of winter wheat IUE, it was found that IUE first increased and then decreased with the change in spring irrigation time, which was consistent with the research results of Liu et al. [56]. The reason was that properly postponing spring irrigation time could promote wheat roots to tie down and improve their absorption and utilization of deep soil water. Optimizing population indexes and canopy structure of winter wheat can promote kernel development [11], thereby increasing yield and water use efficiency. However, if spring irrigation time is too late, the decline rate of tillering will be significantly reduced, and the number of panicles will be reduced, thereby reducing winter wheat yield and leading to the decrease of IWUE [57]. In addition, compared with conventional spring irrigation with second water, wheat yield will be reduced, but IWUE can be increased [58,59]. Wang et al. [60] found that spring irrigation with one water significantly increased IWUE by 72.65~75.71% compared with spring irrigation with second water, which was also confirmed in our previous report [12,61]. In contrast, this study can not only stabilize wheat yield but also improve irrigation water utilization efficiency by adjusting irrigation time under the condition of spring irrigation with one water, which is a water-saving irrigation model suitable for promotion in this region.

## 5. Conclusions

Delayed irrigation for 5 to 10 days at the jointing stage could increase the nitrogen concentration, nitrogen accumulation, silicon concentration, and silicon distribution in the whole plant or different organs, optimize the distribution ratio of nitrogen and silicon in different organs, and realize the improvement of GY. Compared with AJ15 and AJ20, the accumulation of N in the vegetative organs of AJ5 increased by 3.05–23.11% in the flowering stage, the transfer volume of N in the post-flowering vegetative organs increased by 14.09–40.10%, and the N distribution rate in the ear of the mature stage increased by 1.71–6.40%. In the past two years, the GY reached the maximum in AJ10 and AJ5, respectively, and increased by 1.37–6.57% and 22.12–27.21% compared with CK, AJ15, and AJ20, respectively. Therefore, under the condition of spring irrigation, it is suggested that the irrigation time should be 5–10 days after the jointing stage, and the topdressing time should be synchronized with irrigation.

## Figures and Tables

**Table 1:** Precipitation during the winter wheat growing seasons (mm) in 2014–2015 and 2019–2020.

Year	Month									During the Whole Growing Season
	10	11	12	1	2	3	4	5	6	
2014–2015	4.3	15.6	0.0	1.2	9.5	4.5	54.9	64.0	0.0	154
2019–2020	4.2	5.0	2.4	2.0	7.8	6.4	15.8	68.4	6.4	118.4

**Table 2:** N/Si contents and its accumulations in winter wheat at different stages.

Year		Treatment	Content (mg·g^−1^)					Accumulation (kg·hm^−2^)					Accumulation per 100 kg GY (kg)
			JS	HS	AS	FS	MS	JS	HS	AS	FS	MS	
Nitrogen	2015	CK	31.15 b	23.58 b	17.77 a	15.51 ab	15.28 a	166.21 a	215.37 a	226.63 a	255.61 a	297.61 a	3.67
		AJ5	31.80 b	20.83 c	46.69 b	15.01 b	15.04 a	158.39 a	204.8 a	229.69 a	250.09 a	285.37 a	3.44
		AJ10	34.48 a	26.78 a	15.41 c	15.77 ab	14.21 a	165.95 a	204.16 a	229.95 a	255.84 a	290.03 a	3.35
		AJ15	31.76 b	23.37 b	18.36 a	17.34 a	15.98 a	128.7 b	175.92 b	226.05 a	252.08 a	305.96 a	3.59
		AJ20	30.94 b	22.73 bc	16.29 b	15.87 ab	14.83 a	131.35 b	187.14 b	199.59 b	229.17 a	279.22 a	3.4
	2020	CK	18.36 d	14.87 a	13.03 a	10.83 c	12.54 b	82.86 a	133.68 a	165.23 b	168.52 b	208.18 b	4.28
		AJ5	18.88 cd	14.62 b	13.91 a	12.74 b	13.43 a	82.12 a	126.23 b	185.89 a	202.88 a	241.73 a	4.07
		AJ10	19.60 b	14.34 c	13.00 a	12.97 b	13.29 ab	84.06 a	107.88 c	164.60 b	197.12 a	234.89 a	4.01
		AJ15	20.90 a	12.91 d	13.28 a	14.26 a	12.50 b	82.82 a	90.86 d	147.33 b	166.94 b	201.15 b	4.31
		AJ20	19.36 bc	12.41 e	13.20 a	13.32 ab	13.15 ab	75.07 b	74.3 e	152.64 b	164.14 b	201.57 b	4.27
Silicon	2015	CK	8.92 a	15.05 a	16.23 a	18.45 a	18.29 a	47.55 a	137.51 a	206.98 b	304.09 a	356.35 a	4.39
		AJ5	8.37 a	11.08 a	15.27 b	18.14 a	18.43 a	41.71 ab	109.01 a	210.13 b	302.24 a	349.67 ab	4.22
		AJ10	9.73 a	11.37 a	15.51 ab	18.58 a	16.81 a	46.84 a	86.69 a	231.33 a	301.36 a	342.95 ab	3.96
		AJ15	8.44 a	12.38 a	15.27 ab	18.14 a	16.70 b	34.19 b	93.17 a	188.81 c	263.66 a	319.64 b	3.75
		AJ20	7.83 a	13.94 a	15.27 b	18.12 a	17.10 a	33.22 b	114.82 a	187.03 c	261.71 a	321.98 ab	3.92
	2020	CK	7.90 c	8.40 c	9.92 c	12.23 c	13.70 b	35.66 b	75.51 d	125.77 c	190.33 d	227.44 d	4.67
		AJ5	9.03 b	11.08 b	10.83 bc	13.97 bc	14.55 b	39.29 b	95.68 a	144.74 b	222.50 bc	261.78 bc	4.40
		AJ10	10.27 a	10.65 b	13.40 a	18.01 a	17.91 a	44.03 a	80.14 c	169.63 a	273.77 a	316.55 a	5.41
		AJ15	11.03 a	12.27 a	11.95 ab	19.27 a	17.10 a	43.73 a	86.34 b	132.56 bc	225.60 b	275.19 b	5.89
		AJ20	8.17 bc	8.15 c	10.52 bc	15.92 b	15.21 b	31.61 c	48.79 e	121.63 c	196.09 cd	233.16 cd	4.94

Note: JS: jointing stage of winter wheat, HS: heading stage of winter wheat, AS: flowering stage of winter wheat, FS: filling stage of winter wheat, MS: maturity stage of winter wheat. Values within columns followed by the same letter are statistically insignificant at the 0.05 level.

**Table 3:** Distribution of N and Si in different wheat parts.

Year	Stage	Treatment	Percentages of Nitrogen (%)			Percentages of Silicon (%)		
			Stem	Leaf	Ear	Stem	Leaf	Ear
2015	anthesis	CK	23.45 b	64.77 a	11.78 d	29.32 a	53.63 ab	17.04 b
		AJ5	23.39 b	64.39 a	12.22 cd	30.20 a	51.46 b	18.34 b
		AJ10	26.26 a	60.24 b	13.50 b	22.57 b	55.52 a	21.91 a
		AJ15	23.41 b	63.26 a	13.32 bc	23.61 b	54.79 a	21.59 a
		AJ20	26.28 a	58.38 b	15.34 a	22.90 b	55.75 a	21.35 a
	mature	CK	4.98 ab	12.90 a	82.12 b	22.96 a	41.28 b	35.76 a
		AJ5	3.52 c	10.05 a	86.43 a	21.56 ab	42.12 ab	36.32 a
		AJ10	4.58 bc	11.32 a	84.10 ab	19.56 ab	43.89 a	36.55 a
		AJ15	4.85 ab	12.42 a	82.73 ab	19.20 b	43.21 a	37.60 a
		AJ20	6.10 a	12.70 a	81.20 b	21.87 ab	42.95 ab	35.17 a
2020	anthesis	CK	26.97 ab	58.18 ab	14.84 a	14.96 c	74.35 a	10.70 d
		AJ5	26.18 b	54.01 b	19.81 a	22.81 a	68.17 b	9.02 e
		AJ10	27.04 ab	55.75 ab	17.22 a	18.96b	63.59 d	17.46 b
		AJ15	23.60 c	58.94 a	17.47 a	17.60 b	65.84 c	16.55 c
		AJ20	28.40 a	55.61 ab	15.99 a	18.60 b	61.28 e	20.12 a
	mature	CK	3.96 a	13.59 a	82.45 ab	20.12 a	55.85 b	24.04 c
		AJ5	3.93 a	12.74 a	83.32 a	18.50 bc	54.43 c	27.07 b
		AJ10	4.47 a	13.02 a	82.50 ab	18.44 bc	53.67 c	27.89 b
		AJ15	4.51 a	13.61 a	81.88 ab	17.77 c	52.61 d	29.62 a
		AJ20	5.04 a	14.36 a	80.60 b	19.09 b	57.32 a	23.58 c

Note: Values within columns followed by the same letter are statistically insignificant at the 0.05 level.

**Table 4:** The net absorption and transfer of nitrogen and silicon in different parts of wheat from anthesis to maturity stage.

Year	Treatment	NTA (kg·hm^−2^)			SiAAAM (kg·hm^−2^)		
		Stem	Leaf	Ear	Stem	Leaf	Ear
2015	CK	−38.15 bc	−108.38 ab	217.46 a	21.28 a	36.15 a	91.95 a
	AJ5	−43.70 ab	−119.34 a	218.54 a	12.08 a	39.24 a	88.23 a
	AJ10	−47.10 a	−106.19 ab	213.27 a	14.75 a	22.24 b	74.63 a
	AJ15	−38.10 bc	−104.76 b	222.87 a	16.59 a	34.33 a	79.9 a
	AJ20	−35.44 c	−80.91 c	195.95 a	27.13 a	33.95 a	73.85 a
2020	CK	−31.5 ab	−57.47 ab	149.69 ab	26.85 a	33.68 b	41.25 b
	AJ5	−34.07 a	−59.30 a	167.92 a	15.41 b	44.02 ab	57.75 a
	AJ10	−29.22 b	−51.24 bc	168.47 a	26.22 a	62.01 a	58.75 a
	AJ15	−22.02 c	−50.12 c	141.48 b	25.49 ab	57.42 ab	59.58 a
	AJ20	−28.56 b	−46.83 c	140.53 b	21.76 ab	59.15 ab	30.51 b

Note: Values within columns followed by the same letter are statistically insignificant at the 0.05 level. ‘−’ represents the decrease in dry matter quality from the anthesis stage to the maturity stage, and no ‘−’ represents the increase in dry matter quality from the anthesis stage to the maturity stage. NTA: N translocation amount from anthesis to maturity; SiAAAM: Si accumulation amount from anthesis to maturity.

**Table 5:** The significance of the F-value from the analysis of variance of various parameters of winter wheat under different irrigation times and years at various developmental stages.

Parameter	GY	NAAA	NAAM	NTA_stem_	NTA_leaf_	SiAAA	SiAAM
Irrigation duration (I)	5.423 **	13.580 **	1.744 NS	9.912 **	13.577 **	26.583 **	6.844 **
Years (Y)	494.287 **	417.794 **	137.851 **	110.919 **	500.729 **	441.056 **	129.995 **
I × Y	3.526 *	4.829 **	3.108 *	4.681 **	3.841 *	1.750 NS	7.521 **

Note: * and ** denote the significance levels at alpha 0.05 and 0.01 obtained by morality. Significant difference (HSD) test. ‘NS’ denotes non-significance. GY: grain yield; NAAA: N accumulation amount at anthesis; NAAM: N accumulation amount at maturity; NTA_stem_: N translocation amount from stem and sheath; NTA_leaf_: N translocation amount from leaf; SiAAA: Si accumulation amount at anthesis; SiAAM: Si accumulation amount at maturity.

## Data Availability

Data will be made available on request.
